# Occupational stress and associated sources and risk factors among nurses in Gaza strip, Palestine: A cross‐sectional survey

**DOI:** 10.1002/nop2.70004

**Published:** 2024-08-21

**Authors:** Ahmed Hassan Albelbeisi, Sameh Musleh Al‐amoudi, Azadeh Anabri, Hassan Abu Obaid, Fady Alijla, Edris Kakemam

**Affiliations:** ^1^ Medical Services Directorate, College of Health Professions Israa University Gaza Strip Palestine; ^2^ College of Health Professions Israa University Gaza Strip Palestine; ^3^ Department of Public Health, School of Health Yasuj University of Medical Sciences Yasuj Iran; ^4^ School of Public Health Tehran University of Medical Sciences Tehran Iran; ^5^ Faculty of Intermediate Studies Israa University Gaza Strip Palestine; ^6^ Indonesian Hospital, Ministry of Health Gaza Strip Palestine; ^7^ St. John Eye Hospital Gaza Strip Palestine; ^8^ Social Determinants of Health Research Center, Research Institute for Prevention of Non‐Communicable Diseases Qazvin University of Medical Sciences Qazvin Iran

**Keywords:** Gaza strip, nurses, occupational stress, Palestine

## Abstract

**Aim:**

To determine the occurrence of occupational stress among Palestinian nurses, and their associated sources and risk factors.

**Design:**

A cross‐sectional descriptive design.

**Methods:**

A total of 250 registered nurses from eight governmental hospitals, using a convenience sampling method. Data collection were conducted using the 30‐items self‐reported Occupational Stress Scale from December 2022 to March 2023. Descriptive statistics, independent sample *t*‐test, one‐way ANOVA, and multiple linear regression analysis were applied to analysis data.

Data analysis included descriptive statistics, independent sample *t*‐test, one‐way ANOVA, and multiple linear regression analysis.

**Results:**

The prevalence of high occupational stress levels was 64.8% (Mean = 3.9 out of 5). The main sources of stress are too much responsibility and work, understaffing, lack of promotion and recognition, inadequate pay, time pressure, and management style. The results regression analysis demonstrated that male nurses with a Masters or PhD degree and those working in fixed shifts experienced higher occupational stress. Moreover, participants who worked overtime hours were more susceptible to stress.

**Conclusions:**

The research indicates that occupational stress presents a notable challenge for nurses in the Gaza Strip, Palestine. It suggests that in order to alleviate this stress, decision‐makers in healthcare policy and hospital management should prioritize the execution of strategies aimed at addressing the primary stressors and risk factors identified.

**Reporting Method:**

This study adhered to the STROBE guidelines.

**Public Contribution:**

A total of 250 registered nurses were taken part in this study by answering a self‐administered study survey.

## 1 INTRODUCTION

Occupational stress is physical and emotional state that has harmful impacts and consequences from the failure to meet the requirements, resources, and capabilities necessary of a worker (Daniel, 2019). The universal truth is that there can be no health without a workforce. However, in modern decades, concerns about the lack and uneven distribution of qualified health professionals have spread to almost all countries, especially in rural and remote areas (Alluhidan et al., 2020; Karan et al., 2021). Also, occupational stress is a common concern for human resource managers in institutions around the world and is a feature of present business that is becoming more and more prevalent (Nisar & Rasheed, 2020). Therefore, occupational stress is the excessive pressure brought on by an inconsistency between an individual's knowledge, skills, and the requirements of the profession (World Health Organization, 2018).

Occupational stress has a number of undesirable effects on employees including the intention to leave the workplace, burnout, impaired job performance, and poor quality of life (Girma et al., 2021; Kakemam, Kalhor, et al., 2019; Mirzaei et al., 2022). Moreover, workplace stress can have a diversity of damaging effects, including migraines, sleep problems, rage outbursts, worry, sleeplessness, hypertension, and a weak immune system, additionally, it may exacerbate medical problems like depression, obesity, and cardiovascular disease (de Sousa Dantas Rodrigues et al., 2021; Gardner‐Cook, 2022).

Nursing is a profession that has been found to experience high levels of stress and has been recognized as a workplace issue in a number of states (Hegney et al., 2019; Singh et al., 2020). Recent studies conducted among different countries around the world showed that occupational stress among nurses varied from one country to another. In Iran, according to a recent systematic review, the overall prevalence of job stress in Iranian nurses was estimated 37.5% (Isfahani et al., 2021). In Ethiopia, prevalence of job stress in Ethiopian nurses was estimated 47.8% (Werke & Weret, 2023). In Saudi Arabia ranged from 18.8% to 74.3% (Miligi et al., 2019). Several identified factors associated with occupational stress in the nursing field include workload, staff shortages, lack of recognition, inadequate pay, overtime, gender, and nurses working rotating shifts (Anand & Mejid, 2018; Kakemam, Raeissi, et al., 2019; Kwiatosz‐Muc et al., 2018).

Like developing states, Palestinian health institutions still suffer of a shortage of in health professionals, on the basis of the most recent statement (2021), there are about two nurses for every thousand person (Albelbeisi et al., 2021a; Ministry of Heath, 2021). In addition, the majority of governmental employees in the Gaza Strip including nurses since 2007 have not received their full salaries due to siege and since 2008 the population of the Gaza Strip has faced four wars and healthcare professionals including nurses bear the responsibility of dealing with the dead and wounded (Abu‐El‐Noor et al., 2022; Albelbeisi et al., 2021b). All of these factors can contribute to occupational stress among healthcare providers and nurses in particular. To date, there is a dearth of research on occupational stress and associated risk factors among Palestinian nurses. Hence, this study aims to determine the occurrence of occupational stress among Palestinian nurses, and their associated sources and risk factors to help establish health policies that reduce the nurse's stressors.

## 2 METHODS

### 2.1 Design

This cross‐sectional descriptive study conducted from December 2022 to March 2023 in eight governmental hospitals in Gaza Strip, Palestine. Gaza Strip is a small portion of the Occupied Palestinian Territories and is divided into five small governorates with an inhabitant of more than two million (Salem, 2022). The included hospitals have diverse services and units like paediatric, surgery, and neonate.

### 2.2 Participants

The population study consisted of all licensed nurses employed in eight mentioned governmental hospitals. Inclusion criteria consisted of nurses who had at least a diploma degree, employed in the current hospital for at least 12 months, and was fixed nurses. Newly hired nursing staff, part‐time nurses, and nurses working outpatient departments were excluded.

### 2.3 Sample size and sampling method

The Cochran formula was used to compute the sample size, (Snedecor & Cochran, 1989) the projected sample size is 384 cases, (5% a margin of error and 95% a confidence level). Hospitals were assigned a proportional quota based on the nurses' strength. Convenience sampling was employed to recruitment nurses in each hospital.

### 2.4 Measurements

Data were gathered by a self‐questionnaire including two section. First section included eight demographic factors such sex, age, marital status, education level, position (*practical*/*staff*/*head of department*/*supervisor*), work hours (per week), clinical experience, and working shift (fixed/rotating). Occupational stress measured using the 30‐item self‐reported Occupational Stress Scale developed by Mosadeghrad, 2013. After obtaining approval from the questionnaire developer, a cross‐cultural guiding process was used in translating the English version into Arabic (Wild et al., 2009). Ten health experts independently validated the final Arabic version of the questionnaire for face and content validity. A content validity index was measured to assess the relevance of the questionnaire items, and all items were rated as relevant (Yusoff, 2019). In addition, the final Arabic version was piloted among 30 eligible nurses and Cronbach's alpha obtained 0.87 for the overall occupational stress and for the entire sample was 0.95. The final Arabic version questionnaire included 5 domains: duty‐related stressors (9 items), role‐related stressors (3 items), working environment‐related stressors (3 items), organizational policies‐related stressors (10 items), and interpersonal relations related‐stressors (5 items) as well as overall perception of stress. A five‐point scale was used to measure participants' responses (1 = very low, 2 = low, 3 = medium, 4 = high, 5 = very high). Scores of 3 and above were allocated to the high‐stress group and the remaining participants were assigned to the low‐stress group (<3).

### 2.5 Data collection

The data were collected by two researchers in person. Before distributing the questionnaire to the nurses, written informed consent was obtained from each participant. Furthermore, explanations about the objectives of the study and how to complete the questionnaire were provided to them. Because of the nurses' demanding schedules, the researchers typically entrusted the questionnaires to the nurses for overnight completion. Subsequently, the researchers conducted multiple visits to the units throughout the week to retrieve the questionnaires. During the collection process, the researchers meticulously reviewed each questionnaire to confirm its thorough completion. Data collection lasted around 4 months.

### 2.6 Bias

The chance of any selection bias was reduced by collecting data in the morning and evening shifts, voluntary participation of nurses, ensuring the confidentiality of data collected, explaining the aim of the study to participants earlier completing the survey, and giving nurses enough time to do this.

### 2.7 Data analysis

Data analysis was conducted using SPSS version 22.0 (IBM Corp., Armonk, NY, USA). The characteristics of the nurses were described by using descriptive statistics such means, standard deviation (SD), frequencies and percentages. Before testing the hypotheses, the Kolmogorov–Smirnov and Shapiro–Wilk tests were performed for assessing the normality of continuous variables. Therefore, the parametric tests including independent‐ samples *t*‐test and one‐way analysis of variance (ANOVA) were applied to determine differences in means between occupational stress and demographic factors. Regarding ANOVA test, Tukey's post hoc test used when there was significant difference. In addition, a multiple linear regression model was used to identify factors associated with occupational stress. Variables with *p*‐values smaller than 0.05 were included in the regression model. In constructing the model, the normality and independence of the residuals, and collinearity were tested. Normality assumption was assessed by checking Q‐Q plot. Independence of the residuals was evaluated by the Durbin–Watson statistics. Finally, the presence of collinearity was tested by calculating the variance inflation factor (VIF; acceptable level ≤ 5) and the tolerance (acceptable level ≤ 0.1). All tests were conducted at the 0.05 level of statistical significance.

### 2.8 Ethical considerations

This study protocol was approved by the Department of Health Research, Ministry of Health, with ethics code REDACTED. Participation was voluntary and confidentiality of responses was ensured, and a written informed consent form was obtained from subjects. All participants had the right to withdrawal at any time from the study without reason.

## 3 RESULTS

### 3.1 Characteristics of the nurses

Table 1 shows the characteristics of the participated nurses, Of the 389 distributed questionnaires; only 250 nurses completed the survey, yielding a response rate of 64.3%. More than half of respondents (52.0%) were female and around 80% were married. Their age ranged from 20 to 55 years (mean = 33.9, SD = 7.01). Four‐fifths of the nurses had at least a bachelor's degree (80.0%). Respondents' mean of work experience was 9.5 years (SD = 6.4), and about 57% worked as a clinical nurse. Only 30.4% work morning shifts and about half of the nurses' work overtime (>35 h).

**TABLE 1:** Characteristics of participants (*N* = 250).

Factors	Frequency	Percentage
Gender		
Male	120	48.0
Female	130	52.0
Age (years) M ± SD: 33.9 ± 07.01		
20–29	65	(26.0)
30–39	141	(56.4)
≥40	44	(17.6)
Marital status		
Single	53	(21.2)
Married	197	(78.8)
Level of education		
Diploma	50	(20.0)
Bachelor degree	135	(54.0)
Masters or PhD degree	65	(26.0)
Position		
Practical/staff	143	57.2
Head of department	55	22.0
Supervisor	52	20.8
Work hours (per week)		
Normal (≤35 h)	127	(50.8)
Overtime (>35)	123	(49.2)
Clinical experience (years) M ± SD: 09.5 ± 06.4		
<5	76	(30.4)
5–10	75	(30.0)
>10	99	(39.6)
Type of working shift		
Fixed (morning)	74	30.4
Rotating	176	70.6

### 3.2 Stressors of job among nurses

Table 2 shows the mean and standard deviations of the five stress dimensions and the prevalence of high stress among the five dimensions. The mean rating for overall occupational stress was 3.9 out of 5. The mean scores among the five dimensions ranged from 3.16 to 3.46. The highest mean scores value reported with ‘organizational policies stressors’ (Mean = 3.46; SD = 0.78) and the lowest with ‘Duty stressors’ and ‘Role stressors’ (M = 3.16; SD = 0.75), (M = 3.16; SD = 1.04) respectively. The major sources of occupational stress were too much responsibility and staff shortages (85.2%), lack of recognition and promotion prospects (84.6%), inadequate pay (82.4%), time pressure (84.0%), too much work (80.0%), and management style (80.0%). In all, 64.8% of the participating nurses stated that the nursing job was a very stressful job. Three‐quarters (75.2%) of the participating nurses informed that they suffer from high‐stress due to institutional rules.

**TABLE 2:** scores for stress dimensions and prevalence of high stress.

Scales/items	*n*	%^a^	Mean	SD
*Duty stressors*	**147**	**58.8**	**3.16**	**0.75**
Job identity	187	74.8	3.00	0.99
Needs more conscious for doing tasks	116	46.4	2.48	1.14
Lack of coordination between the job and your abilities	97	38.8	2.34	1.16
Too much work	200	80.0	3.46	1.04
Time pressure	205	82.0	3.55	1.06
Insufficient regular breaks at work	191	76.4	3.44	1.13
Low decision latitude	175	70.0	3.38	1.15
Too much responsibility	213	85.2	3.66	0.99
Given responsibility without the authority to take decisions	173	69.2	3.15	1.14
*Role stressors*	**157**	**62.8**	**3.16**	**1.04**
Role ambiguity	160	64.0	3.13	1.24
Role contradiction	175	70.0	3.09	1.15
Conflicting demands	180	72.0	3.27	1.13
*Working environment stressors*	**175**	**70.0**	**3.42**	**0.98**
Inappropriate working condition	182	72.6	3.41	1.18
Inadequate resources	199	79.6	3.49	1.08
Hazardous situation	183	73.2	3.35	1.16
*Organizational policies stressors*	**188**	**75.2**	**3.46**	**0.78**
Inadequate pay	206	82.4	3.82	1.16
Lack of recognition and promotion prospects	212	84.6	3.87	1.12
Lack of job security	189	75.6	3.45	1.16
Limited or no access to training	183	73.2	3.32	1.06
Unusual controls (too much or too little supervision or control)	193	77.2	3.32	1.02
Management style	200	80.0	3.35	1.05
Changes imposed from managers	186	63.4	3.19	1.08
Policies and regulations	197	78.8	3.26	1.06
Staff shortages	213	85.2	3.76	1.10
Work shifts	178	71.2	3.28	1.21
*Interpersonal relations stressors*	**163**	**65.2**	**3.24**	**0.91**
Bullying and harassment behaviour from managers	178	71.2	3.23	1.21
Bullying and harassment behaviour from co‐workers	140	56.0	2.79	1.11
Bullying and harassment behaviour from customers	190	76.0	3.32	1.12
Lack of management support	198	79.2	3.49	1.16
Discrimination at work	184	73.6	3.37	1.20
*Overall perception of job stress*	**162**	**64.8**	**3.29**	**0.78**

*Note*: The bolds indicate the scores for overall scale and subscales.

^a^ Numbers and proportions of the nurses who rated their job stress 3 and above 3.0, respectively.

### 3.3 Participants' occupational stress means scores with demographic variables

Table 3 shows a comparison of occupational stress means scores with demographic variables, significant differences were reported in occupational stress and nurses' gender, level of education, work hours (per week), clinical experience (years), and type of working shift, with a *p* < 0.05.

**TABLE 3:** Participants' occupational means stress scores with demographic variables.

Factors	Mean (SD)	*p*‐Value
Gender		
Male	3.53 (0.68)	<0.001^a^ ^,^ *
Female	3.06 (0.79)	
Age (years)		
20–29	3.27 (0.82)	.412^b^
30–39	3.33 (0.76)	
≥40	3.16 (0.79)	
Marital status		
Single	3.26 (0.94)	0.780
Married	3.29 (0.74)	
Level of education		
Diploma	3.01 (0.68)	<.001^b^ ^,^ *
Bachelor degree	3.19 (0.82)	
Masters or PhD degree	3.66 (0.64)	
Position		
Practical/staff	3.10 (0.69)	.085^b^
Head of department	3.31 (0.77)	
Supervisor	3.42 (0.85)	
Work hours (per week)		
Normal (≤35 h)	3.10 (0.77)	<0.001^a^
Overtime (>35)	3.47 (0.75)	
Clinical experience (years)		
<5	3.05 (0.82)	.003^b^ ^,^ *
5–10	3.48 (0.78)	
>10	3.32 (0.70)	
Type of working shift		
Fixed (morning)	2.99 (0.79)	<0.001,^a^ ^,^ *
Rotating	3.41 (0.74)	

* *p* < 0.01.

^a^ Independent‐samples *t*‐test.

^b^ One‐way ANOVA.

The outcomes demonstrated that male nurses have higher occupational stress score than female (*p* < 0.001). Nurses who worked more than 35 h per week have higher occupational stress scores than nurses worked 35 h or less per week (*p* < 0.001). Nurses who worked shifts (morning/evening/night) than nurses who worked morning shifts only (*p* < 0.001). After applying Tukey post hoc test, there were a statistically significant difference observed, in level of education, nurses with diploma certificate having a significantly lower stress compared to the nurses who had bachelor's and postgraduate certificates (*p* < 0.001), in clinical experience (years), nurses with less than 5 years of experience having a significantly lower stress compared to the nurses who had experience between 5 and 10 years (*p* = 0.003).

### 3.4 Factors associated with occupational stress

Table 4 shows the outcomes of multiple linear regression analysis of predictor variables for occupational stress. There was no evidence of multicollinearity, as assessed by tolerance values >0.1 and variance inflation factor <5. There was independence of residuals, as assessed by a Durbin‐Watson statistic of 1.21. The model had an *R* ^2^ of 0.215 and adjusted *R* ^2^ of 0.199. Male nurses had higher stress than female nurses (*β* = 0.25, 95% CI 0.07 to 0.45, *p =* 0.006). Nurses with a Masters or PhD degree experienced higher occupational stress than those with Bachelor degree and Diploma (*β* = −0.34, 95% CI −0.56 to −0.12, *p =* 0.002), and (*β* = −0.41, 95% CI −0.68 to −0.14, *p* = 0.003) respectively. Nurses who worked overtime hours were more susceptible to stress than nurses who did not (*β* = −0.24, 95% CI −0.42 to −0.06, *p* = 0.010). Participants working in fixed shifts had significantly lower stress than those in rotating shifts (*β* = −0.37, 95% CI −0.56 to −0.17, *p* < 0.001).

**TABLE 4:** Regression analysis of factors associated with occupational stress.

Factors	*β*	95% CI		*p*‐Value
		Lower	Upper	
Intercept	3.72	3.46	3.98	<0.001*
*Gender* (reference = female)	0.25	0.07	0.45	0.006*
*Work hours* (reference = > 35 h)	−0.24	−0.42	−0.06	0.010**
*Clinical experience* (reference ≥ 10 years)				
<5 years	−0.18	−0.50	−0.05	0.31
5–10 years	0.09	−0.13	0.31	0.415
*Type of working shift* (reference = Rotating)	−0.37	−0.56	−0.17	<0.001*
*Level of education* (*reference = Masters or PhD degree*)				
Diploma	−0.41	−0.68	−0.14	0.003*
Bachelor degree	−0.34	−0.56	−0.12	0.002*

*Note*: *R* ^2^ = 0.215; adjusted *R* ^2^ = 0.199; *F* = 13.36; *p* < 0.001; *p < 0.01; **p < 0.05.

## 4 DISCUSSION

To date, there is a dearth of research on occupational stress and associated sources and risk factors among Palestinian nurses. Based on the findings, nurses reported a high level of occupational stress (64% of nurses). The results of our study were roughly comparable to the studies that have been conducted in Ethiopia (56.3%) (Anand & Mejid, 2018), Iran (78.4%) (Kakemam, Kalhor, et al., 2019), Saudi Arabia ranged from 18.8% to 74.3% (Miligi et al., 2019), and Jordan (50.1%) (Al‐Amer et al., 2022). The difference may be due to the variety of tools used and the difference in sample size from one study to another, in addition to the availability of occupational health and safety practices in some countries and their absence in others. Furthermore, a possible explanation of high prevalence of the stress among Palestinian nurses could be related to shortage of nurses' staff number, lack of adequate capacity and readiness to provide effective healthcare, lack of safety sense in clinical area, and working in an environment of instability and constant risk (Albelbeisi et al., 2021b; PCBS, 2018; Qaraman et al., 2022; Veronese et al., 2020).

Our study revealed that the main sources of stress are due to too much responsibility and staff shortages, lack of recognition and promotion prospects, inadequate pay, time pressure, too much work, and management style. A possible explanation for these results is that Palestinian health institutions still suffer of a shortage of in health professionals, there are about two nurses for every thousand person (Albelbeisi et al., 2021a; Ministry of Heath, 2021). In addition, the majority of governmental employees in the Gaza Strip including nurses since 2007 have not received their full salaries due to siege and since 2008 the population of the Gaza Strip has faced four wars and healthcare professionals including nurses bear the responsibility of dealing with the dead and wounded (Abu‐El‐Noor et al., 2022; Albelbeisi et al., 2021b). Similar results were found in Iran, Kuwait, and Poland (Kakemam, Kalhor, et al., 2019; Kwiatosz‐Muc et al., 2018; Vickers, 2017).

Regression analysis showed higher occupational stress associated with female nurses, nurses with a postgraduate or bachelor's degree, nurses who worked overtime, and nurses working rotating shifts. Male had higher stress than female, these results are inconsistent with previous studies carried out in Iran, Japan and West Africa, and West Bank that found female nurses were more stressed at the workplace than male (Ayivi‐Guedehoussou, 2016; Gadirzadeh et al., 2017; Jaradat, 2017; Yada et al., 2014). In the West Bank (Northern governorates of Palestine) study, the results demonstrated that female workers reported higher levels of occupational stress than males (Jaradat, 2017). A possible explanation could be related to nurses' roles that include childcare and domestic service responsibilities more suited to females in Eastern societies and to the belief that males are better suited to management roles (Lantz, 2008; Tlaiss, 2013).

Nurses with a postgraduate or bachelor's degree experienced higher occupational stress than those with a diploma, these findings are in agreement with previous studies carried out in Uganda and Iran (Gadirzadeh et al., 2017; Opoku Agyemang et al., 2022). A study carried out among Ethiopian nurses demonstrated that obtaining a university degree rather than a diploma was identified as a significant factor associated with the intention to leave a nursing job (Tsegaw et al., 2022). Literature shows that more educated health professionals have higher expectations for their careers and they will be more upset if these expectations are not met (Al‐Makhaita et al., 2014; Ayivi‐Guedehoussou, 2016). In contrast with our findings, a study conducted among Iranian nurses reported that nurses with lower educational attainment reported higher levels of stress (Kakemam, Raeissi, et al., 2019).

The findings revealed that nurses who worked overtime were more susceptible to stress than nurses who did not. In line with this, Iranian and Ugandan studies have shown that working hours are associated with work‐related stress in the nursing field and burnout (Kakemam, Kalhor, et al., 2019; Nabirye et al., 2011; Vandevala et al., 2017). In the Palestinian context, nurses in governmental hospitals work 36 hours per week, and around half of the participating nurses (49.2%) reported working overtime, nurses are allowed to work in both governmental and no‐ governmental health organizations at the same time which facilitates increased overtime. Considering total hours worked per week is important in managing occupational stress for those with many jobs.

Nurses working in rotating shifts had significantly higher stress than those in fixed shifts. A possible explanation for these results is that rotating nurses experienced difficulties implementing and maintaining daily activities such as physical activity, due to fatigue, an inability to participate in socially‐formed activities, increased health risks due to reduced sleep duration (Chiang et al., 2022; Torquati et al., 2019). This result is consistent with previous studies conducted in Ethiopia, West Bank, and Taiwan (Jaradat, 2017; Torquati et al., 2019; Werke & Weret, 2023). A study conducted among Taiwan nurses demonstrated that fixed‐morning‐shift nurses had significantly reduced perceived stress compared with rotating‐shift nurses. Furthermore, among rotating‐shift nurses, fixed‐evening‐ or fixed‐night‐shift nurses had lesser perceived stress than non‐fixed‐rotating‐shift nurses (Torquati et al., 2019). Therefore, since the nursing profession is compulsory that needs rotating shifts, nurse managers are advised to fix the rotating shift of nurse.

### 4.1 Limitations

This study has several limitations: the use of a convenience selection method and a self‐reported questionnaire that can lead to various types of bias (social desirability and recall bias). Future research should consider employing probability sampling methods such as simple random sampling, stratified sampling, cluster sampling, and systematic sampling to recruit nurses. In addition, in addition, not assessing other variables that may have affected stress levels such as household conflicts, labor conflicts, and daily stress which may impact nurses' occupational stress. Therefore, it is recommended that the researchers explore the impact of other possible drivers and confounding variables on occupational stress in future studies.

## 5 CONCLUSION

The current study establishes that occupational stress was a significant problem among nurses in Gaza Strip, Palestine. The main sources of stress are too much responsibility and work, understaffing, lack of promotion and recognition, inadequate pay, time pressure, and management style. The present study demonstrates that occupational stress is a noteworthy issue experienced by nurses in the Gaza Strip, Palestine. Key stressors include excessive workload and responsibilities, understaffing, lack of promotional opportunities and acknowledgment, inadequate compensation, time constraints, and management approach. It is suggested that in order to alleviate occupational stress, health policymakers and hospital administrators should prioritize the implementation of strategies aimed at mitigating the primary stressors and identified risk factors. These interventions encompass enhancing workplace conditions, establishing resilience‐promoting initiatives for nurses, implementing job advancement and enrichment programs, revising employee work schedules, increasing staffing levels, and re‐evaluating compensation packages.

## AUTHOR CONTRIBUTIONS

A.H.A. and E.K. designed and conducted the study, performed the analysis and drafted the manuscript. S.A., H.O. and F.A. advised on the study design, facilitated data collection and revised the manuscript. S.A., H.O. and F.A. helped in data collection. A.H.A., E.K. and A.A. interpretation of data and revised the manuscript. All authors reviewed and approved the manuscript.

## FUNDING INFORMATION

The authors have not received any funding.

## CONFLICT OF INTEREST STATEMENT

The authors declare that they have no competing interests.

## ETHICS STATEMENT

This study protocol was approved by the Department of Health Research, Ministry of Health, with ethics code 1133842. Participation was voluntary and confidentiality of responses was ensured, and a written informed consent form was obtained from subjects.

## Data Availability

The datasets used and/or analysed during the current study are available from the corresponding author on reasonable request.
